# Phenolic compound profile of probiotic (*Lacticaseibacillus rhamnosus* LR5) fortified vegetable tablet and probiotic survival in the simulated gastrointestinal tract

**DOI:** 10.1038/s41598-022-04874-z

**Published:** 2022-01-19

**Authors:** Saeid Jafari, Krongkan Thongmat, Isaya Kijpatanasilp, Paramaporn Kerdsup, Phisut Naknaen, Malai Taweechotipatr, Kitipong Assatarakul

**Affiliations:** 1 Department of Food Technology, Faculty of Science, Chulalongkorn University, Bangkok, 10330 Thailand; 2 Division of Biotechnology and Agricultural Product, Faculty of Agricultural Product Innovation and Technology, Srinakharinwirot University, Nakhon Nayok, 26120 Thailand; 3 Division of Food Science and Nutrition, Faculty of Agricultural Product Innovation and Technology, Srinakharinwirot University, Nakhon Nayok, 26120 Thailand; 4 Department of Microbiology, Faculty of Medicine, Srinakharinwirot University, Bangkok, 10110 Thailand

**Keywords:** Applied microbiology, Nutrition

## Abstract

The objectives of this research were to study the changes of phenolic compounds in vegetable (yellow *VS* green) tablets with/without probiotics (*Lacticaseibacillus rhamnosus* LR5) supplementation by using high performance liquid chromatography and probiotic survivability through the simulated gastrointestinal tract. The green vegetable tablets with/without probiotics had a greater (*p* ≤ 0.05) phenolic content compared to the yellow ones. There were no significant differences of most phenolic compound contents between probiotic-supplemented vegetable tablets and non-probiotic supplemented ones (*p* > 0.05). The contents of ferulic acid, epicatechin, tannic acid and rutin for both vegetable tablets tended to decrease through passing the stomach (1 and 2 h) and small intestine (2 and 4 h), however, the content of catechin in the yellow vegetable tablets tended to increase. The results also showed that the survival of *Lacticaseibacillus rhamnosus* LR5 slightly decreased through the simulated gastrointestinal tract. The vibrations from FTIR appeared in the wave length of 4000–3100, 3000–2800 and 1652–1545 cm^−1^, which accounted for the change in the N–H bonds of the amine group, changes in the structure of fatty acids and the change of carbonyl groups, respectively. This work highlighted the opportunity of application of probiotics in food products; especially non-dairy foods for consumer with dairy allergy.

## Introduction

The Food and Agriculture Organization (FAO) of the United Nations and the World Health Organization (WHO) defined probiotics as “live microorganisms that, when administered in adequate amounts, confer a health benefit on the host”^1^. The development of probiotic supplements has been around for a long time and continues which is mainly because of many health benefits of probiotics on reducing the risk of colorectal cancer and modulation of immune system. However, the most of probiotic supplements sold are milk-based products (e.g., yogurt), not many elderly people consume them because of being lactose intolerance or allergic^2^. The development of a non-milk-based probiotic supplement is an option to help people get more probiotics. Among different strains of probiotics, *Lacticaseibacillus rhamnosus* is one of the most studied species and isolated from the intestinal tract of healthy humans. It has been claimed for several health benefits including prevention of gastrointestinal infection, prevention of intestinal diarrhea, and prevention of necrotizing enterocolitis^2^. In addition, improvement of human's natural defenses by *Lacticaseibacillus rhamnosus* has been also documented^3^. *Lacticaseibacillus rhamnosus* LR5 is a common probiotic strain with a variety of food application and it is commercially available. Supplementing vegetable tablets with probiotic is a new health alternative on the market. Furthermore, different fruits or vegetables contain different types of phenolic compounds which could inhibit and stimulate the growth of certain microorganisms and can reduce the risk of certain diseases making vegetable tablets more and more popular today^4^. The presence of phenolic compounds can enhance probiotic survival in different foods^5^. Some studies showed that probiotics could tolerate gastric acid, use phenolic compounds in foods (especially fruits and vegetables) as substrates, and increase their survival and functionality while passing through the digestive tract resulting in the reduced risk of colon cancer^6^.

In vitro simulations of human digestion have become popular because they are less labor intensive, less costly and they are not subject to the same ethical constraints as in vivo studies. Because of their controlled conditions, reproducibility, and ease of sampling, they are ideal for mechanistic studies^7^. However, there is no information in the literature about the phenolic contents of probiotic fortified vegetable tablets in the simulated digestive system. Therefore, the objectives of this study were to evaluate the effect of the addition of probiotic strain *L. rhamnosus* LR5 on the phenolic compound profile in yellow and green vegetable tablets submitted to in vitro simulated gastrointestinal conditions (simulated early and middle digestive tract) and on probiotic survival.

## Materials and methods

### Preparation of vegetable tablets and supplementing with probiotics

Two kinds of vegetable tablets (yellow *VS* green) were used in this study. Respectively, twelve and three kinds of vegetables were used for yellow and green vegetable tablets (Table 1). All vegetables used in this study were obtained from private vegetable farming in Thailand which were collected in accordance with and permission under the Thai national standard on Natural Raw Material Collection. All vegetables were vacuum dried and fine powdered. Then, each kind of dried vegetable (yellow and green) were mixed with lyophilized powder *Lacticaseibacillus rhamnosus* LR5 (Brenntag Thailand Co., Ltd) to contain at least 10^6^ CFU/g according to the probiotic food product standard by the Ministry of Public Health, Thailand. The yellow vegetable tablet was commercially available product (Chiangmai Bioveggie Co., Ltd., Thailand) while the green one was developed by our research group. After mixing, samples were pressured (0.01 hPa) by Hammer tablets with a single punch press machine (Sinopham, TDP-6). The round, convex tablet weighed 250 mg/tablet for both yellow and green vegetable tablets. The samples were kept in aluminum foil bag for 24 h at ambient temperature before the analysis. The initial population of probiotics in vegetable tablet was approximately 10^8^ CFU/g.

**Table 1:** Ingredients of yellow and green vegetable tablets (mg/tablet).

Yellow tablet			Green tablet		
Ingredient	Amount	%	Ingredient	Amount	%
Beet Root	10	4	Carrot	20	8
Chinese spinach (Puay Leng)	0.5	0.2	Pumpkin	50	20
Celery	10	4	Asparagus	130	52
Japanese Pumpkin	103	41.2	Dibasic calcium phosphate anhydrous (Emcompress®)	40	16
Broccoli	0.5	0.2	Colloidal silicon dioxide	3	1.2
Cherry tomatoes	10	4	Magnesium stearate	7	2.8
Carrot	20	8			
Coriander	6	2.4			
Purple cabbage	0.5	0.2			
Sweet pepper	8	3.2			
Parsley	0.5	0.2			
Japanese spring onions	31	12.4			
Dibasic calcium phosphate anhydrous (Emcompress®)	40	16			
Colloidal silicon dioxide	3	1.2			
Magnesium stearate	7	2.8			
Total	250	100	total	250	100

*Dibasic calcium phosphate anhydrous (Emcompress®) (as filler), Colloidal silicon dioxide (as glidant), Magnesium stearate (as lubricant).

### The simulation of the early and middle gastrointestinal tract

The simulation of the early and middle gastrointestinal tract was conducted according to Michida, et al.^8^. In brief, vegetable tablets (2 g) were dissolved in distilled water (40 mL) (adjust the pH to 2 with 6 M concentrated hydrochloric acid). Then, for the early and middle simulation of the gastrointestinal tract, pepsin protease (105.6 mg) was added and incubated in Eppendorf Thermomixer C (Model 5382, USA) at 37 °C for 2 h (sampling every 1 h). After incubation, pancreatin from porcine pancreas (2 mL) and bile extract porcine (2 mL) were incubated (pH = 7.4) in Eppendorf Thermomixer C (Model 5382, USA) at 37 °C for 4 h (sample collected every 2 h). Finally, the samples were collected into an Eppendorf tube (1.5 mL), centrifuged (10,000 × g for 5 min at 4 °C) and filtered through 0.45 μm membrane filter for the further analysis by HPLC as above mentioned.

### Analysis of phenolic compounds in vegetable tablets with/without probiotics

The samples were kept in aluminum foil bag for 24 h at ambient temperature before the analysis of phenolic compound profile. The initial phenolic compounds were analyzed for yellow and green tablets, yellow and green formulations, both with and without probiotic supplementation using high performance liquid chromatography (HPLC, Agilent 1100 Series, Germany). The analysis of phenolic compounds in vegetable tablets when passing through the primary and central digestive systems was also performed by HPLC (Agilent 1100 Series, Germany). In brief, aliquots were collected at the beginning (0 h) and after each gastrointestinal phase (stomach: after 1 h and 2 h; small intestine: after 2 h and 4 h) for analysis of phenolic compounds, probiotic survival and FITR. The aliquots were filtered through a filter (0.45 µm) before injection into the HPLC machine. Then, the sample solution as well as standard were injected to HPLC. The phenolic contents were calculated with respect to the delay of the chromatogram from the sample obtained with the standard substance chromatogram. All standard reagents were purchased from Sigma-Aldrich (St. Louis, MO, USA). The phenolic contents were reported as part per million (ppm) units.

### Probiotic survivability analysis

The samples (aliquots) were collected from simulated gastrointestinal experiment for determination of probiotic survival after the different gastrointestinal phases. The population of *L. rhamnosus* LR5 was determined according to AOAC^9^ using a plate technique in MRS agar culture medium. In brief, 5 g probiotic-supplemented vegetable pellet samples were dissolved in a 45 mL 0.1% (w/v) peptone solution then serialized and diluted. The sample solution (1 mL) was poured into the Petri dish and mixed well. The incubation was performed at 37 °C for 48 h and the results were reported in log CFU/g.

### Investigating probiotic membrane changes with Fourier transform infrared spectroscopy (FTIR)

Transformation of probiotic cell membranes by Fourier transform infrared spectroscopy (FTIR) was conducted according to Gandhi and Shah^10^ methods. In brief, probiotic-supplemented vegetable tablets passed through the early and middle digestive systems were centrifuged (10,000 × g for 10 min at 4 °C), rinsed with distilled water twice and put in the calcium fluoride (CaF_2_) optical plate. The FTIR analysis was done with wavelength 4000–1000 cm^−1^, 20 times of scans and 4.0 cm^−1^ of resolution.

### Statistical analysis

The data were analyzed using a Completely Randomized Design (CRD) and all experiments were performed in triplicate. The mean was compared with Tukey's HSD (honestly significant difference) test by SPSS version 22 (Statistical Package for Social Sciences) program at 95% confidence level (*p* ≤ 0.05) to compare the significant differences.

## Results and discussion

### Phenolic contents in vegetable tablets with/without probiotics

Phenolic compounds are secondary metabolites found in most plants with health beneficial effects including antioxidant and anti-inflammatory properties^11^. Many studies reported the strongly positive correlation between phenolic compound content and antioxidant activity^12^. Daily consumption of fruit and vegetable has been widely recommended because phenolic compound are associated with a decreased risk of several chronic diseases such as cardiovascular disease, obesity and cancer^13^. The present study compared changes of phenolic compound profile of the commercial vegetable tablet (yellow tablet) and developed vegetable tablet (green tablet) after simulated gastrointestinal digestion. A Japanese pumpkin was selected and used in yellow tablet as the highest amount (41.2%) which was responsible for yellow color appearance of tablet whereas green asparagus (52%) was accounted for green color appearance of green tablet. The initial phenolic contents of vegetable tablets (yellow *VS* green) with or without probiotic supplementation are shown in Table 2. The phenolic contents tested were; gallic acid, catechin, ferulic acid, epicatechin, tannic acid, chlorogenic acid, rutin, quercetin and apigenin. The green vegetable tablets with/without probiotics had a greater (*p* ≤ 0.05) phenolic content compared to yellow vegetable tablets. Most of phenolic compound contents in probiotic-supplemented vegetable tablets were also recorded as no significant differences compared to non-probiotic supplemented ones (*p* > 0.05). The catechin content in both vegetable tablets was the highest among the other phenolic compounds except for green vegetable tablets with probiotics. However, the green tablets had the greater (*p* ≤ 0.05) catechin value as compared with the yellow ones. The apigenin contents in both vegetable tablets were the lowest phenolic contents among the others. The apigenin ​​values were (0.24 and 0.69 ppm) *VS* (14.09 and 1.31 ppm) in yellow and green vegetable tablets with/without probiotic supplementation, respectively. The quercetin content was not detected in all samples as a result of either too high sensitivity of the analyzer or quercetin in the sample being too little, which could not be detected when compared with other phenolic compounds.

**Table 2:** Initial phenolic compound contents (ppm) of vegetable tablets with/without probiotics.

Phenolic compound contents	Yellow tablet	Green tablet	Yellow tablet + probiotics	Green tablet + probiotics
Gallic acid	3.60 ± 0.06^c^	20.51 ± 1.53^b^	4.66 ± 0.68^c^	127.10 ± 0.39^a^
Catechin	36.05 ± 1.14^c^	3059.74 ± 102.07^a^	36.15 ± 1.48^c^	857.35 ± 42.73^b^
Ferulic acid	13.29 ± 0.32^b^	28.34 ± 0.82^a^	13.76 ± 0.49^b^	27.84 ± 1.53^a^
Epicatechin	4.32 ± 0.43^b^	1497.70 ± 99.50^a^	2.04 ± 0.02^b^	1539.20 ± 68.14^a^
Tannic acid	1.62 ± 0.14^b^	62.37 ± 1.99^a^	1.52 ± 0.04^b^	58.47 ± 1.85^a^
Chlorogenic acid	6.13 ± 0.48^b^	1009.61 ± 94.81^a^	5.83 ± 0.58^b^	1079.80 ± 88.75^a^
Rutin	13.18 ± 0.82^b^	235.57 ± 14.59^a^	16.85 ± 0.37^b^	216.41 ± 0.75^a^
Quercetin	ND	ND	ND	ND
Apigenin	0.24 ± 0.02^d^	14.09 ± 0.78^a^	0.69 ± 0.11^c^	1.31 ± 0.05^b^

*mean ± standard deviation.

ND: not detected.

^a,b,c,d^Different superscript letters (within same row) indicate significant differences (*p* ≤ 0.05).

**Table 3:** Phenolic compound contents (ppm) of yellow vegetable tablets with/without probiotics when passing through the simulated early and middle digestive system.

Phenolic compound contents	Probiotics	Stomach		Small intestine	
		1 h	2 h	2 h	4 h
Gallic acid	With	4.43 ± 0.04^b^	4.44 ± 0.09^b^	5.54 ± 0.53^a^	5.75 ± 0.53^a^
	Without	4.48 ± 0.26^c^	4.08 ± 0.18^d^	4.83 ± 0.11^b^	5.67 ± 0.13^a^
Catechin	With	39.44 ± 0.65^c^	39.37 ± 1.34^c^	46.83 ± 0.98^b^	49.04 ± 0.35^a^
	Without	43.78 ± 3.59^ab^	44.02 ± 3.46^ab^	45.83 ± 0.40^a^	48.00 ± 3.53^a^
Ferulic acid	With	13.76 ± 0.37^a^	13.67 ± 0.42^a^	1.88 ± 0.52^b^	1.63 ± 0.15^b^
	Without	14.31 ± 0.53^a^	1.44 ± 0.16^b^	1.33 ± 0.08^b^	1.78 ± 0.29^b^
Epicatechin	With	1.85 ± 0.02^a^	1.83 ± 0.03^a^	1.73 ± 0.16^b^	1.60 ± 0.05^b^
	Without	4.74 ± 0.02^a^	2.84 ± 0.21^b^	0.73 ± 0.08^c^	1.57 ± 1.08^b^
Tannic acid	With	1.58 ± 0.13^b^	1.47 ± 0.03^c^	1.71 ± 0.07^a^	1.61 ± 0.02^b^
	Without	1.73 ± 0.16^a^	1.58 ± 0.03^b^	0.54 ± 0.07^d^	0.87 ± 0.33^c^
Chlorogenic acid	With	5.70 ± 0.19^a^	5.79 ± 0.14^a^	4.07 ± 0.28^b^	4.25 ± 0.02^b^
	Without	6.63 ± 0.50^a^	5.06 ± 0.02^b^	4.36 ± 0.38^c^	5.11 ± 0.98^b^
Rutin	With	16.34 ± 0.49^a^	16.53 ± 0.49^a^	1.10 ± 0.03^b^	1.74 ± 0.64^b^
	Without	18.71 ± 0.45^a^	15.26 ± 0.01^b^	1.26 ± 0.08^c^	1.56 ± 0.86^c^
Quercetin	With	ND	ND	ND	ND
	Without	ND	ND	ND	ND
Apigenin	With	0.65 ± 0.09^a^	0.69 ± 0.06^a^	ND	ND
	Without	0.23 ± 0.01^c^	0.45 ± 0.05^b^	0.59 ± 0.30^b^	0.78 ± 0.06^a^

* mean ± standard deviation.

ND: not detected.

^a,b,c,d^Different superscript letters (within same row) indicate significant differences (*p* ≤ 0.05).

**Table 4:** Phenolic compound content (ppm) of green vegetable tablets with/without probiotics when passing through the simulated early and middle digestive systems.

Phenolic compound content	Probiotics	Stomach		Small intestine	
		1 h	2 h	2 h	4 h
Gallic acid	With	25.76 ± 0.67^b^	27.24 ± 2.42^b^	39.14 ± 0.29^a^	39.35 ± 1.39^a^
	Without	22.92 ± 0.95^d^	27.52 ± 2.03^c^	34.96 ± 0.08^b^	39.56 ± 1.70^a^
Catechin	With	3233.20 ± 49.41^b^	3389.90 ± 2.31^a^	587.37 ± 1.78^c^	562.90 ± 30.26^c^
	Without	3245.28 ± 66.52^a^	3356.60 ± 49.46^a^	526.12 ± 14.13^b^	539.21 ± 3.24^b^
Ferulic acid	With	28.21 ± 0.39^a^	10.91 ± 0.22^b^	4.27 ± 0.01^c^	4.80 ± 0.27^c^
	Without	27.47 ± 0.65^a^	28.37 ± 0.42^a^	4.36 ± 0.12^b^	4.81 ± 0.25^b^
Epicatechin	With	1670.30 ± 37.64^b^	1724.7 ± 37.89^a^	15.65 ± 1.44^d^	27.15 ± 1.68^c^
	Without	1677.03 ± 28.84^b^	1727.63 ± 33.74^a^	27.32 ± 0.92^c^	18.40 ± 0.08^d^
Tannic acid	With	69.00 ± 5.16^b^	84.51 ± 8.41^a^	44.63 ± 1.46^c^	36.45 ± 1.91^d^
	Without	71.98 ± 0.95^a^	73.38 ± 0.26^a^	32.98 ± 0.87^c^	35.86 ± 1.07^b^
Chlorogenic acid	With	1225.70 ± 37.98^b^	1363.9 ± 1.59^a^	107.99 ± 5.51^d^	139.26 ± 5.01^c^
	Without	1267.29 ± 20.78^b^	1348.63 ± 23.22^a^	102.95 ± 1.61^c^	95.07 ± 3.19^d^
Rutin	With	256.22 ± 9.91^a^	117.53 ± 3.22^b^	73.64 ± 3.28^c^	53.61 ± 2.04^d^
	Without	256.95 ± 8.88^a^	269.13 ± 8.65^a^	48.76 ± 3.62^b^	47.02 ± 2.63^b^
Quercetin	With	ND	ND	ND	ND
	Without	ND	ND	ND	ND
Apigenin	With	0.83 ± 0.08^b^	1.61 ± 0.67^a^	1.64 ± 0.24^a^	1.55 ± 0.41^a^
	Without	5.64 ± 0.35^a^	5.95 ± 0.45^a^	1.09 ± 0.06^c^	1.65 ± 0.12^b^

*mean ± standard deviation.

ND: not detected.

^a,b,c,d^Different superscript letters (within same row) indicate significant differences (*p* ≤ 0.05).

In the recent years, several raw materials have been extensively explored to produce novel non-dairy functional foods in which vegetables have been proposed as a novel suitable carrier medium for probiotics^14^. In the current study, probiotic supplementation had no significant (*p* > 0.05) effect on most phenolic compound contents of vegetable tablet. However, a significantly higher content of apigenin was observed in yellow formulation with probiotic supplementation. In addition, catechin and apigenin contents of green vegetable table with probiotic were significantly (*p* ≤ 0.05) lower whereas gallic acid content was significantly (*p* ≤ 0.05) higher than those of without probiotic formulation. In our study, the catechin content was one of the most phenolic compounds found in the yellow and green vegetable tablets. These results are consistent with Ruidavets, et al.^15^ who reported that the concentration of catechin in plasma was three-fold higher in diet with vegetable as compared with non-vegetable diet. Catechin is a phenolic compound found mostly in green tea and other plants. The catechin is an antioxidant capable of eliminating free radicals. Phenolic compounds (e.g., catechins) have been reported to be antioxidants, however their effectiveness depend on the quantity, duration of administration and interaction with other elements of food^16^. Moreover, there are reports about the other health benefits of catechin on other diseases such as cardiovascular disease and diseases related to the brain and liver disease^17^. The connection with sugar is typical of the epicatechin molecule in fruits and vegetables. Such acid-bonding resulted in the epicatechin molecules closely resembling those of the standard HPLC analyzers, enabling increased amounts of epicatechin to be detected. A high value of chlorogenic acid was observed in the current study among the green vegetable tablets with and without probiotic supplementation (1079.80 and 1009.61 ppm, respectively). Chlorogenic acid is classified as hydroxycinnamic acid with caffeic acid and quinic acid in the basic structure^18^. Currently, there are extensive researches into the health benefits of chlorogenic acid which could be found in some plants such as apple, carrot, coffee bean, kiwi, plum, potato, tea and tomato, etc.^19^. Some of the health benefits of chlorogenic acid are anti-oxidation, anti-inflammatory, anti-cancer and anti-diabetes properties^18^.

### Phenolic contents in vegetable tablets when subjected to simulated early and middle digestive system

In vitro simulations of human digestion have become popular because they are less labor intensive, less costly and they are not subject to the same ethical constraints as in vivo studies. Because of their controlled conditions, reproducibility, and ease of sampling, they are ideal for mechanistic studies^7^. The contents of ferulic acid, epicatechin, tannic acid and rutin for both vegetable tablets tended to decrease through passing the stomach (1 and 2 h) and small intestine (2 and 4 h) small intestine (Tables 3 and 4), however, the content of catechin in the yellow vegetable tablets tended to increase. The catechin contents in the yellow vegetable tablets with probiotics were 39.44, 39.37, 46.83 and 49.04 ppm when passing through the stomach (1 and 2 h) and small intestine (2 and 4 h) small intestine, respectively. However, the apigenin content had the lowest content (0.65 and 0.69 ppm) in the yellow vegetable tablets with probiotics through the stomach (1 and 2 h) as compared with the other phenolic compounds. The changes in the phenolic contents in the green vegetable tablets with and without probiotic supplementation showed that gallic acid tended to increase when passing through the early and middle digestive tract. However, the remaining phenolic compounds (catechin, ferulic acid, epicatechin, tannic acid, chlorogenic acid, rutin, quercetin and apigenin) tended to decrease. These changes in the phenolic contents of the vegetable tablets (with/without probiotic) when passing through the early digestive tract (stomach) and the middle part of the small intestine could be due to the acidic environment as well as bile salts and gastric juice from the pancreas which result in various structural changes in phenolic compounds^20^ Furthermore, probiotic metabolism could result in alteration of phenolic compound content^21^. The phenolic compounds are reactively metabolized due to hydrolysis and oxidative reaction during gastrointestinal tract digestion with probiotic activity resulting in the transformation of phenolic compounds which completely different from the initial phenolic compounds. The phenolic compounds (caffeic acid, chlorogenic acid, ferulic acid and gallic acid) can influence protein hydrolysis which results in a decrease in the performance of digestive enzymes^22^. However, the phenolic compound can react with different enzymes and digestive juices in the gastrointestinal tract resulting in inhibition of biological reactions. These are associated to the prevention of human chronic diseases^23^.

In the present study, the chlorogenic acid may have been degraded to its derivatives after gastrointestinal digestion resulting a decrease trend when passing through the simulated early and middle digestive system. This result is in agreement with the report from Rueda et al.^24^ who showed that phenolic compounds generally decreased after simulated in vitro gastrointestinal digestion. In addition, this present work reveals that acid and alkaline hydrolysis during the simulated digestion have a significant effect on the stability of phenolic compounds. The greatest decrease of phenolic compound was found in ferulic acid and this might be due to the structural modifications during the digestion process^25^. Structural modifications of phenolic compound during simulated digestive tract may possible be an important mechanism of an alteration of phenolic compound content which may reduce bioaccessibility^26^. Probiotics could be able to influence on phenolic compound contents due to various reactions leading to the formation of phenolic derivatives^27^.

### Probiotic (*L. rhamnosus* LR5) survivability in vegetable tablets when subjected to the simulated early and middle digestive system

**Figure 1:**
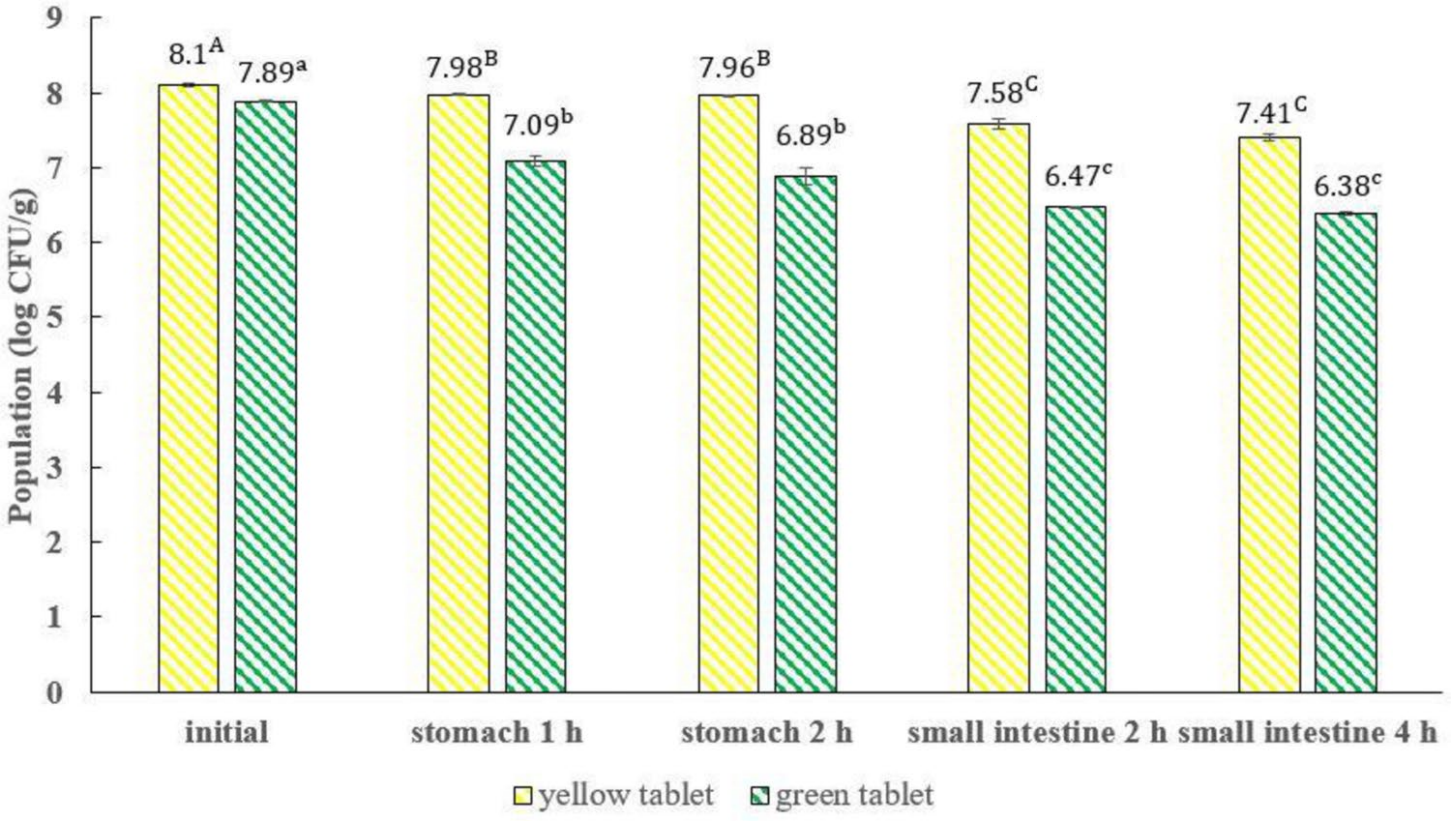
Survivability of *L. rhamnosus* LR5 in probiotic-supplemented vegetable tablets when passing through the simulated early and middle digestive tract. A, B, C ; Different superscript letters (yellow tablet) indicate significant differences (*p* ≤ 0.05). a, b, c ; Different superscript letters (green tablet) indicate significant differences (*p* ≤ 0.05).

The survival of probiotics through the different parts of the digestive tract is vital to the health benefits of consumers. The survivability of *L. rhamnosus* LR5 in probiotic-supplemented yellow vegetable tablets was higher than that of the green ones (Fig. 1). The initial *L. rhamnosus* LR5 in the yellow vegetable tablets was 8.10 log CFU/g which moderately decreased to 7.98, 7.96, 7.58 and 7.41 (log CFU/g) when passing through the early and middle digestive systems. The initial *L. rhamnosus* content of the probiotic-supplement green vegetable tablets was also 7.89 log CFU/g. The *L. rhamnosus* LR5 population tended to decrease to 7.09, 6.89, 6.47 and 6.38 log CFU/g when passing through the early and middle digestive systems. The lower *L. rhamnosus* LR5 in the vegetable tablets while passing through the early and middle digestive tract could be due to the low gastric pH (2.5–3.5). Probiotics have shown to have a low acid tolerance^28^. In the small intestine, bile salts act as a detergent to reduce the surface tension of fat particles which results in antimicrobial activity by degrading the microbial cell membranes of *Lactobacillus* sp.^29^. However, despite the lower *L. rhamnosus* LR5 content through the middle digestive tract, it was more than 10^6^ CFU/g, which is large enough to be beneficial to health^5^. One reason for this phenomenon could be due to the fact that probiotics use phenolic compounds containing in vegetable tablets as substrates, increasing their survivability^5^. The reduction of probiotic survival in green vegetable t ablet with probiotics was higher than in yellow vegetable tablet with probiotics. This may due to the fact that the difference of formulations between yellow and green vegetable tablets with probiotics might influence the survival of probiotics under in vitro gastrointestinal simulation^24,28,30^.

Probiotic survival throughout the digestive system in terms of acidic and gastric environment imparts the health benefit to consumer. It has been reported that *L. rhamnosus* HN001 was formulated in cheesecake and its population remained higher than 10^6^ CFU/g throughout the storage at − 20 °C and 4 °C with positive effect on sensory properties^31^. In addition, recent study on health effect of *L. rhamnosus* has been reported. A short-term consumption of fermented milk containing *L. rhamnosus* SD11 demonstrated that it provided a beneficial effect on oral health by reducing salivary levels of Streptococcus mutans (prevention of oral cavity) with no side effects as a result of producing antimicrobial protein and it can be considered as potential strains contributing to a boost of human’s oral health^32^. An application of *L. rhamnosus* was also found in non-dairy foods such as juices. Study conducted by Andrade et al.^33^ showed that inulin added to fermented and non-fermented guava juices improved the survival of *L. rhamnosus* ATCC 7469. The viability of this strain was about 8 log CFU/mL throughout the refrigerated storage. Recent studies have shown that phenolic compounds may regulate gut microbial population by pathogen inhibition and beneficial bacterial’s growth enhancement. This leads to a prebiotic function resulting in mutual relationship between phenolic compounds and probiotics or protective effect of phenolic compounds on probiotic survival^30^.

### Probiotic transformation by Fourier transform infrared spectroscopy (FTIR)

**Figure 2:**
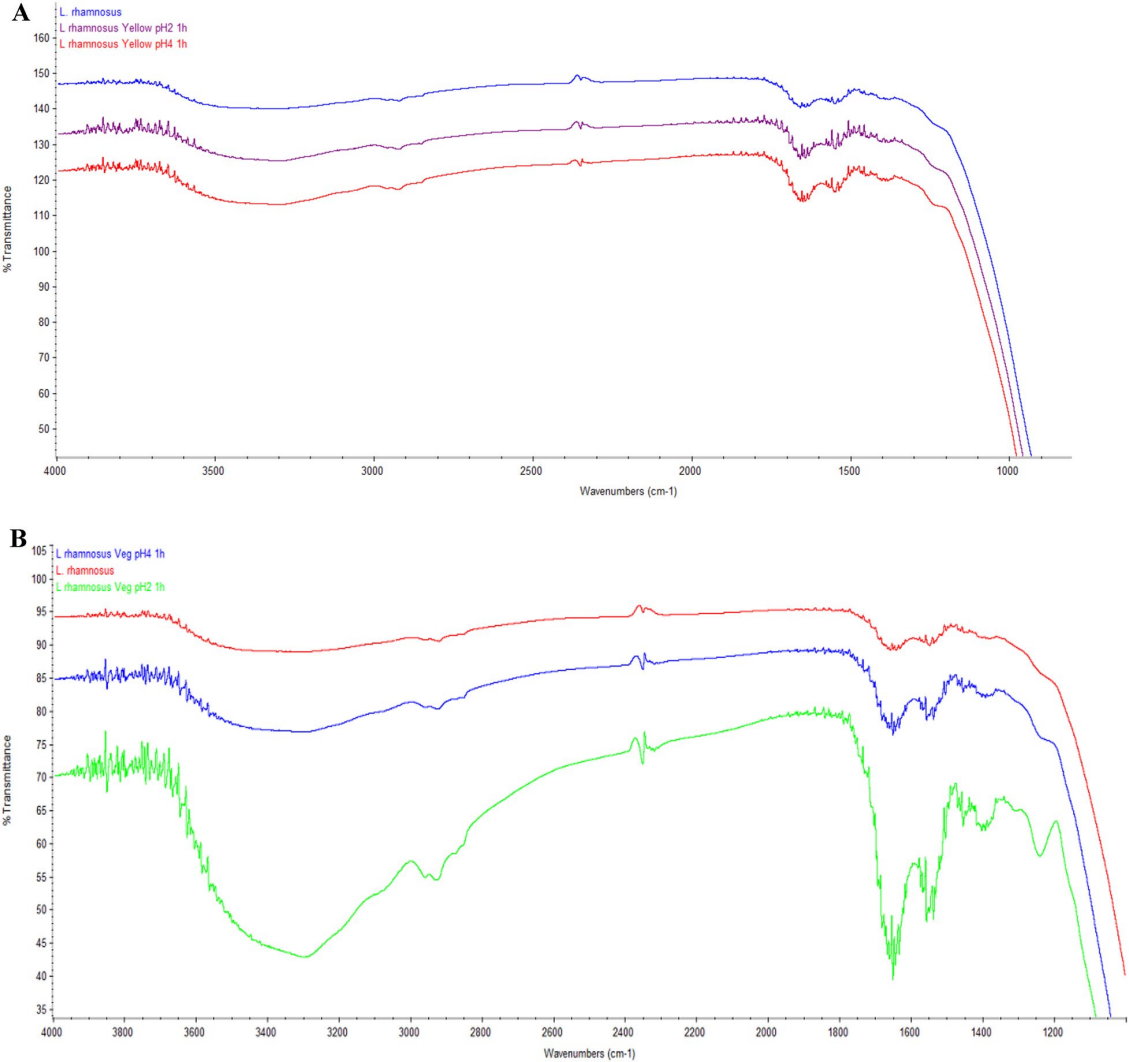
FT-IR spectra of *L. rhamnosus* LR5 in the stomach with yellow (**A**) and green (**B**) vegetable tablets.

The fourier transform infrared spectroscopy (FTIR) technique is commonly used in the analysis of transformations and examining biochemical molecules. In other word, FTIR technique has been known as a tool for the analysis of the microbiological quality of foods^34^. A little biochemical conversion of *L. rhamnosus* LR5 in gastric acidity was observed in the yellow vegetable tablets *VS* the green ones (Fig. 2A and B), From the FTIR transformation analysis, oscillations were found in the wavelength range 4000–3100 cm^−1^, indicating the elongation of the NH amine group bonds and the OH bonds. It was found that green vegetable tablets with probiotics at pH 2 showed a large elongation of the NH bond which leads to protein degradation. In addition, oscillations in the region of 3000–2800 cm^−1^ showed elongation of asymmetric CH_3_, asymmetric CH_2_ and symmetric CH_3_ in membrane fatty acids which changes the structure of fatty acids from lyotropic gel to liquid crystalline and *Lactobacillus* sp. can be found to change fatty acids in an acidic state. A large change in green vegetable tablets *VS* yellow ones was found when *L. rhamnosus* LR5 was in acidic conditions. This is possible due to the difference in formulation of ingredients resulting in difference in phenolic compound contents^30^. The oscillations in the wavelength range 1850–1750 cm^−1^ represents the change in nucleic acids, the wavelength between 1700 and 1300 cm^−1^ is the change in lipids and proteins, and 1400 cm^−1^ wavelength extending carboxyl group of amino acids and fatty acids. Our results showed the changes in every wavelength of *L. rhamnosus* LR5 in acidic conditions with green vegetable tablets. The more change could result in a denaturation of the bonds and conformation changes of macromolecules such as protein and fatty acid leading to a less resistance to acidic conditions since *L. rhamnosus* LR5 in green vegetable tablets had a lower gut survival rate than that of yellow vegetable ones. It might also be unfavorable condition of probiotic for rapid adaptation to acidosis^10^.

## Conclusions

This present study demonstrated that no significant differences of most of phenolic compounds were observed after probiotic supplementation. The phenolic contents of vegetable tablets with/without probiotics decreased when passing through the simulated early and middle digestive systems except for the gallic acid and catechin content. The survivability of *L. rhamnosus* LR5 of all samples tended to decrease after passing through the simulated early and middle digestive systems, however, the content of *L. rhamnosus* LR5 in both vegetable tablets was greater than 10^6^ CFU/g which is sufficient to promote the health of the consumer. According to FTIR analysis, the oscillations was found in the wavelength range of 4000–3100 cm^−1^ indicating a transformation of specific molecular bonds resulting in an alteration in cell surface of probiotic. This could considerably influence the probiotic survival. In conclusion, this research could be used as a guideline for the development of vegetable tablet with probiotic fortification for health-conscious consumer. However, further study such as probiotic viability during the storage is still necessary.
